# Pharmacokinetics of Miltefosine in Children and Adults with Cutaneous Leishmaniasis

**DOI:** 10.1128/AAC.02198-16

**Published:** 2017-02-23

**Authors:** María del Mar Castro, Maria Adelaida Gomez, Anke E. Kip, Alexandra Cossio, Eduardo Ortiz, Adriana Navas, Thomas P. C. Dorlo, Nancy Gore Saravia

**Affiliations:** a Centro Internacional de Entrenamiento e Investigaciones Médicas, Cali, Colombia; b Division of Pharmacoepidemiology & Clinical Pharmacology, Utrecht University, Utrecht, the Netherlands; c Department of Pharmacy & Pharmacology, Antoni van Leeuwenhoek Hospital, Amsterdam, the Netherlands; d Department of Pharmaceutical Biosciences, Uppsala University, Uppsala, Sweden

**Keywords:** miltefosine, pharmacokinetics, cutaneous leishmaniasis, children, intracellular miltefosine

## Abstract

An open-label pharmacokinetics (PK) clinical trial was conducted to comparatively assess the PK and explore the pharmacodynamics (PD) of miltefosine in children and adults with cutaneous leishmaniasis (CL) in Colombia. Sixty patients, 30 children aged 2 to 12 years and 30 adults aged 18 to 60 years, were enrolled. Participants received miltefosine (Impavido) at a nominal dose of 2.5 mg/kg/day for 28 days. Miltefosine concentrations were measured in plasma and peripheral blood mononuclear cells by liquid chromatography-tandem mass spectrometry of samples obtained during treatment and up to 6 months following completion of treatment, when therapeutic outcome was determined. Fifty-two patients were cured, 5 pediatric patients failed treatment, and 3 participants were lost to follow-up. Leishmania (Viannia) panamensis predominated among the strains isolated (42/46; 91%). Noncompartmental analysis demonstrated that plasma and intracellular miltefosine concentrations were, overall, lower in children than in adults. Exposure to miltefosine, estimated by area under the concentration-time curve and maximum concentration, was significantly lower in children in both the central and intracellular compartments (*P* < 0.01). Leishmania persistence was detected in 43% of study participants at the end of treatment and in 27% at 90 days after initiation of treatment. Clinical response was not dependent on parasite elimination. *In vitro* miltefosine susceptibility was similar for Leishmania strains from adults and children. Our results document PK differences for miltefosine in children and adults with cutaneous leishmaniasis that affect drug exposure and could influence the outcome of treatment, and they provide bases for optimizing therapeutic regimens for CL in pediatric populations. (This study has been registered at ClinicalTrials.gov under identifier NCT01462500.)

## INTRODUCTION

Children with cutaneous leishmaniasis (CL) present several diagnostic and therapeutic challenges compared to adults with CL. These include greater pathogenicity of Leishmania infection in children (1), propensity for facial lesions (2), and subtherapeutic drug exposure due to interruption or abandonment of treatment resulting from the logistical demands of parenteral administration and/or higher elimination rates of antimony (3). There is no recommended treatment for CL in neonates and infants <2 years of age (4). Limited therapeutic options and increased incidence of CL among children compel the development of more effective treatment for this vulnerable population.

Oral miltefosine is well tolerated and generally efficacious against Old World visceral leishmaniasis (VL) and CL (5–10), although efficacy varies geographically and variable susceptibility to miltefosine of Leishmania species causing CL has been suggested by *in vitro* evaluations (11, 12). Miltefosine was shown to be noninferior to meglumine antimoniate in the treatment of pediatric CL in Colombia (13), and its efficacy was corroborated by clinical trials in populations where Leishmania (Viannia) guyanensis and Leishmania (Viannia) braziliensis are endemic in Brazil (8, 9). Nevertheless, pharmacokinetics (PK) modeling of miltefosine in Indian and Nepalese children with VL, a systemic disease characterized by hypoalbuminemia and hypergammaglobulinemia, which can affect drug pharmacokinetics, showed that linear milligrams-per-kilogram dosing resulted in underdosing and that treatment failure was linked to lower drug exposure (14, 15). PK data for miltefosine in children with CL are unavailable and are needed to define drug exposure and concentration-effect relationships in this clinically and physiologically distinct presentation of leishmaniasis.

Since Leishmania parasites are intracellular pathogens, the drug concentration within host cells is critical to the direct antimicrobial effect. PK are generally determined in plasma under the assumption that systemic drug exposure is proportional to and predictive of exposure in target tissues/cells (16). However, analyses of intracellular concentrations of other antimicrobials have shown that this assumption is not consistently upheld and that substantial differences between systemic and intracellular concentrations can occur (17). Drug concentration in the target tissue is a key determinant of therapeutic response, influencing the elimination of infection and selection of resistant organisms. Neither intracellular concentrations of any antileishmanial drug nor their relationships to plasma concentrations or PK parameters associated with parasitological and clinical responses are available.

We report the results of an open-label pharmacokinetic trial of miltefosine in children and adults with CL. Plasma and intracellular PK were determined and clinical and parasitological responses evaluated to provide PK bases for optimizing use of miltefosine in pediatric CL.

## RESULTS

### Study participants and Leishmania species.

**FIG 1:**
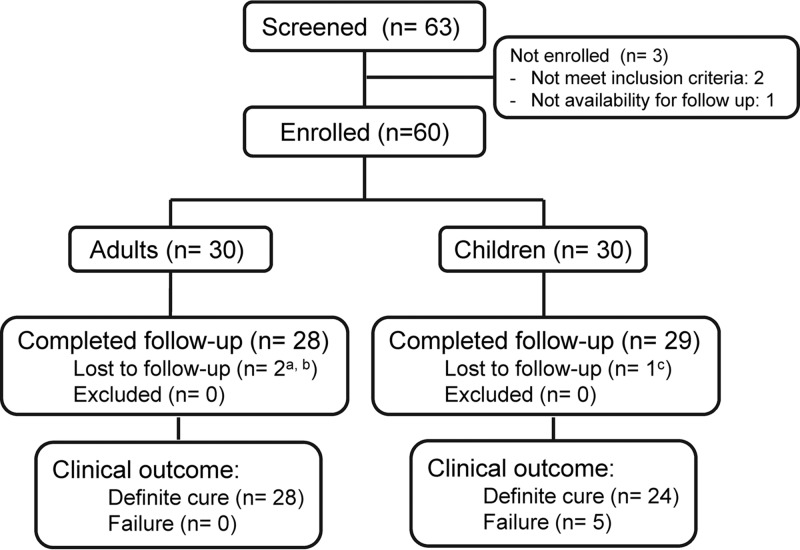
Participant enrollment and follow-up. a, both declared lost at day 90; b, one of two lost patients did not attend the end of treatment visit but attended the day 60 visit; c, declared lost at the day 120 visit.

Sixty-three patients were assessed for eligibility; two did not meet inclusion criteria based on clinical laboratory analysis, and one declared unavailability for follow-up. Among the 60 enrolled participants, two adults and one child were lost to follow-up (Fig. 1). In both study groups (Table 1), participants were predominantly males of Afro-Colombian descent. No statistical differences were found between children and adults in number, location, and diameter of lesions. However, the median duration of disease was significantly shorter and ulcerative lesions were more frequent in children. Leishmania (Viannia) species were isolated from 83% of adults and 70% of children. *L*. (*V*.) panamensis was the most prevalent species, at 95% (20/21) in children and 88% (22/25) in adults. *L*. (*V*.) braziliensis was isolated from one child and two adults.

**TABLE 1:** Baseline characteristics of the study groups

Characteristic	Result for:		*P* value
	Children (*n* = 30)	Adults (*n* = 30)	
Sociodemographic			
Age (yr), mean (SD)	8.16 (2.58)	33.53 (8.32)	
Male gender, no. (%)	18 (60)	14 (46.67)	0.3^*a*^
Ethnicity, no. (%)			0.76^*a*^
Afro-Colombian	22 (73.33)	23 (76.67)	
Mestizo	8 (26.67)	7 (23.33)	
Study site, no. (%)			0.57^*a*^
Cali	8 (26.67)	10 (33.33)	
Tumaco	22 (73.33)	20 (66.67)	
Clinical			
Wt (kg), mean (SD)	26.22 (7.62)	70.84 (11.73)	
No. of lesions, median (IQR)	2 (1–3)	1 (1–2)	0.39^*b*^
Time evolution of older lesion (mo), median (IQR)	1 (1–2)	2 (2–3)	0.007^*b*^
Hemoglobin (g/dl), mean (SD)	12.7 (0.84)	13.71 (1.57)	0.002^*c*^
Albumin (g/dl), mean (SD)	4.50 (0.26)	4.57 (0.27)	0.33^*c*^
Dose (mg/kg/day), mean (SD)	2.27 (0.16)	2.11 (0.32)	0.02^*c*^
Lesions (*n* = 118)			
Type, no. (%)			0.003^*d*^
Ulcer	54 (85.71)	33 (60.00)	
Plaque	7 (11.11)	19 (34.55)	
Other	2 (3.17)	3 (5.45)	
Location, no. (%)			0.35^*a*^
Face-neck	11 (17.46)	6 (10.91)	
Trunk	9 (14.29)	9 (16.36)	
Arms	21 (33.33)	26 (47.27)	
Legs	22 (34.92)	14 (25.45)	
Satellite lesions, no. (%)			0.69^*a*^
Yes	13 (20.63)	13 (23.64)	
No	50 (79.37)	42 (76.36)	
Adenopathy associated, no. (%)			0.3^*d*^
Yes	3 (4.76)	6 (10.91)	
No	60 (95.24)	49 (89.09)	
Maximum diameter of largest lesion (mm), median (IQR)	30 (23.8–40)	32.5 (26–46)	0.48^*b*^
Area of largest lesion (mm^2^), median (IQR)	548.99 (412.33–1,193.80)	659.73 (351.85–1,481.26)	0.72^*b*^
Leishmania strains, no. (%)			0.51^*c*^
*L*. (*V*.) panamensis	20 (66.67)	22 (73.33)	
*L*. (*V*.) braziliensis	1 (3.33)	2 (6.67)	
L. mexicana	0	1 (3.33)	
Not isolated	9 (30.00)	5 (16.67)	

a χ^2^ test.

b Mann-Whitney U test.

c *t* test.

d Fisher exact test.

### Pharmacokinetics.

**FIG 2:**
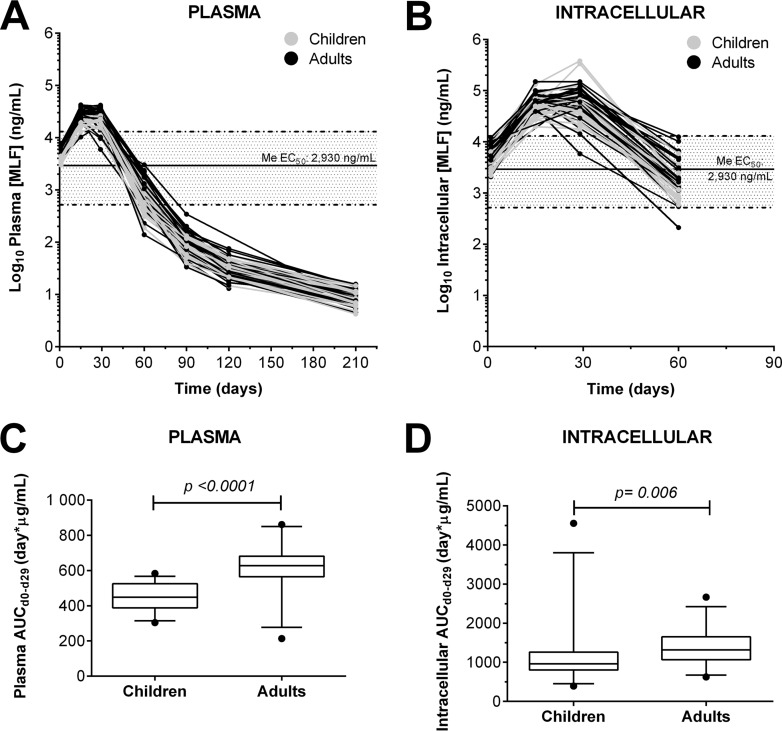
Concentration-time curves of miltefosine in plasma and PBMC samples. (A and B) Miltefosine concentrations measured in plasma (A) and PBMCs (intracellular) (B) from samples obtained from children (*n* = 30) and adults (*n* = 30) throughout the course of treatment and up to 6 months of follow-up. (C and D) Plasma (C) and intracellular (D) areas under the concentration-time curve. Box plots show median values and 5th to 95th percentiles. Solid lines represent the median EC_50_ and dashed lines represent the minimum and maximum EC_50_ reported for Leishmania (Viannia) clinical strains from a similar patient cohort (19).

Miltefosine concentrations in plasma were detectable for up to 6 months following completion of treatment (Fig. 2). Intracellular miltefosine was measurable for up to 1 month after completion of treatment; thereafter, concentrations fell below the limit of detection (4 ng/ml) (18).

Intracellular miltefosine concentration-time profiles followed those in plasma. Intracellular accumulation of miltefosine was evident, resulting in a 2-fold higher maximum concentration (*C*_max_) than in plasma (Table 2). The median plasma and intracellular concentrations during treatment (Fig. 2) exceeded the median 50% effective concentrations (EC_50_) for strains from a previously reported patient cohort (19). Plasma and intracellular *C*_max_ and overall exposure (area under the concentration-time curve from day 0 to 29 [AUC_d0–29_] and AUC_d0–∞_) were lower (*P* < 0.01) in children than in adults (Fig. 2; Table 2). The determination of intracellular AUC_d0–∞_ and elimination half-life (t_1/2_)was precluded by unavailability of ≥3 data points from the elimination phase. Although no significant differences in time to *C*_max_ (*T*_max_) or elimination half-life were observed among groups, miltefosine plasma concentrations declined >40% between days 15 and 29 for 6 adults and one child, and this was reflected in the paired intracellular concentrations.

**TABLE 2:** Summary of plasma and intracellular pharmacokinetic parameters

Parameter	Value for:				*P* value^*b*^
	Children (*n* = 30)		Adults (*n* = 29)^*a*^		
	Median	Range	Median	Range	
Plasma					
*C*_max_ (μg/ml)	22.7	17.0–29.3	31.9	17.2–42.4	1.421e−06**
*T*_max_ (days)	27.8	13.9–28.0	16.0	13.8–28.1	0.21
*t*_1/2_ (days)	37.1	7.4–47.0	34.4	9.5–46.15	0.07221
AUC_d0–d29_ (μg · day/ml)	448	304–583	628	213–861	4.484e−07**
AUC_d0–∞_ (μg · day/ml)	652	438–832	880	427–1,206	5.645e−06**^c^
Intracellular					
*C*_max_ (μg/ml)	55.6	19.8–382	71.5	40.0–150	0.006168*
*T*_max_ (days)	23.2	13.0–28.0	27.5	13.8–30.0	0.4236
AUC_d0–d29_ (μg · day/ml)	964	393–4,552	1316	625–2,667	0.006794

a One patient was excluded from the noncompartmental analyses because of insufficient data points.

b *, *P* < 0.01; **, *P* < 0.001 (by Mann-Whitney U test unless otherwise indicated).

c By Student *t* test.

### Compliance with therapy.

All visits were attended by >95% of patients, with exception of visit 7 (day 120), which 90% (53/59) of participants attended (see Table S1 in the supplemental material). Although the sampling window established for visits was ±7 days, the median window of attendance was 2 to 3 days for any visit. Adherence to treatment based on diary and pill count was similar for children and adults (children, median of 100% and range of 90% to 100%; adults, median of 100% and range of 89% to 100%). Adherence by two participants who did not return their diary (one adult and one child) could be assessed only by pill count, indicating ≥96.4% drug use. Additionally, one adult presented a discrepancy between reported doses and pill count; the adherence based on pill count was 76%. The previously mentioned decline in miltefosine concentration for six adult patients and one pediatric patient is discordant with the corresponding diary and pill count and could indicate nonadherence that was not perceived by self-reporting. Notably, these seven patients were cured.

### Pharmacodynamics and parasitological response.

**FIG 3:**
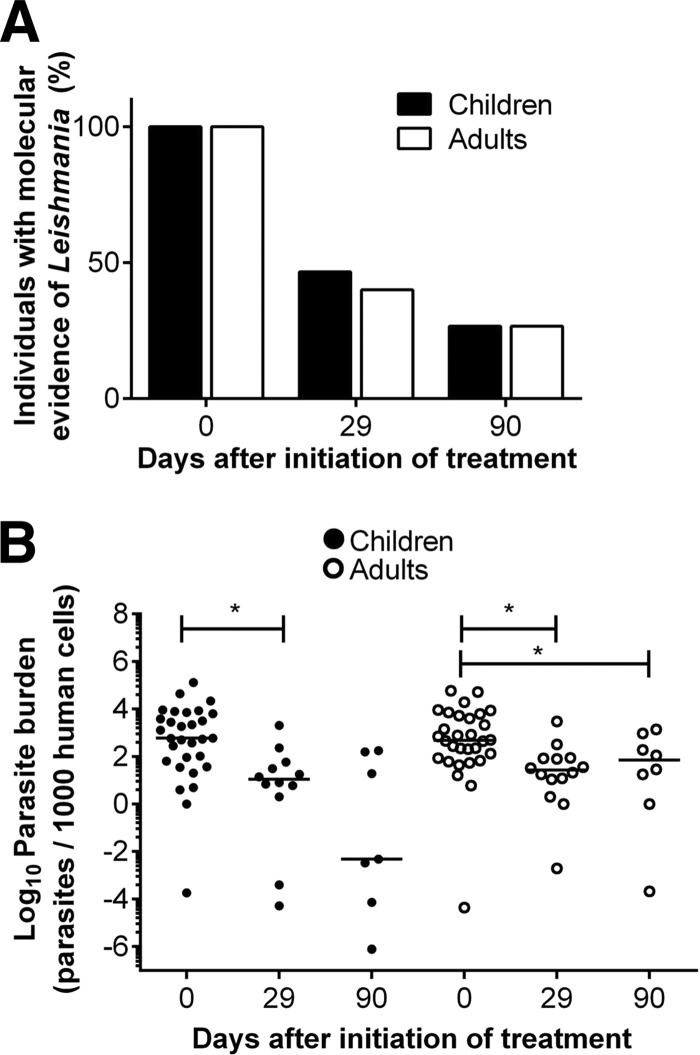
Dynamics of Leishmania persistence after end of treatment in children and adults. Qualitative assessment of Leishmania persistence determined by kDNA positivity or amplification of the 7SLRNA transcript (A) and parasite loads (B) in children and adults in lesions or lesion scars at the end of treatment (day 29) and 90 days after beginning of treatment are shown. Leishmania persistence is presented as the percentage of individuals with at least one kDNA- or 7SLRNA-positive sample. Quantitative values for parasite loads are presented as the number of parasites quantified for every 1,000 human cells.

The presence and viability of Leishmania in lesions before treatment was demonstrated in all patients. Parasite detection declined to 44% (26/59 patients) at end of treatment (EoT) based on aspirate or swab samples obtained from active or apparently healed lesions, and it diminished to 27% (16/59) at day 90 (Fig. 3A). Among patients with detectable parasites in lesions at EoT, the parasite load had nevertheless significantly decreased (Fig. 3B). Neither the proportion of individuals presenting molecular evidence of Leishmania persistence nor parasite loads differed between children and adults at any time point (Fig. 3A and B). No correlation was observed between miltefosine exposure (intracellular and plasma EoT concentrations, *C*_max_, or AUC_d0–29_) and measurable parasite loads at the lesion site. Likewise no relationship between parasite clearance and clinical outcome was evident.

**FIG 4:**
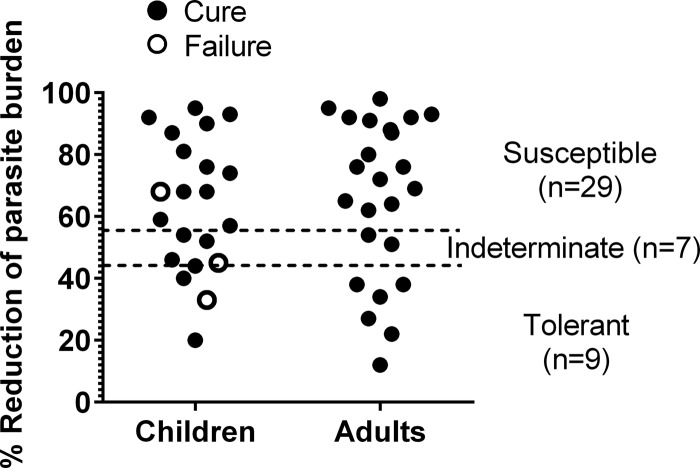
Susceptibility of isolated Leishmania strains to miltefosine, showing the reduction of intracellular parasite burden at the discriminatory concentration of miltefosine (16 μM). The cutoff thresholds (dashed lines) and indeterminate zone were defined based on previously described receiver operating characteristic (ROC) curves (19). For children versus adults, *P* = 0.38; for susceptibility versus treatment outcome, *P* = 0.15.

Among strains isolated from study participants, 64.4% (29/45) were susceptible to miltefosine *in vitro*, 20% (9/45) were tolerant, and 15.6% (7/45) were classified as indeterminate. No differences were observed between the susceptibility profiles of strains isolated from children and adults (Fig. 4). Leishmania strains isolated from patients who clinically responded to treatment presented a range of *in vitro* susceptibility, with a median reduction of parasite burden of 70.5% (interquartile range [IQR], 53% to 89%). There was no apparent relationship between parasite drug susceptibility and clinical outcome.

### Therapeutic response.

Definite cure, defined at 6 months after end of treatment, was achieved in all adults (28/28) and 82.7% of children (24/29). Despite the higher frequency of therapeutic failure in children, this difference was not statistically significant (*P* = 0.052). Notably, treatment failure occurred only among children ≤7 years of age. No significant differences in time to cure were found between study groups (Table 3). The logistic regression model used to explore the relationship between therapeutic response, PK parameters, and clinical and parasitological variables, revealed that age was an independent risk factor for therapeutic failure; for every additional year of age, the odds of failure decreased (odds ratio [OR] = 0.30; 95% confidence interval [CI], 0.09 to 0.97).

**TABLE 3:** Treatment response by follow-up visit and age group

Treatment response by follow-up visit	No. (%)		*P* value
	Children (*n* = 30)	Adults (*n* = 30)	
End of treatment (day 29)			
Apparent cure	1 (3.33)	1 (3.33)	0.74^*a*^
Improvement	29 (96.67)	27 (90.00)	
No change	0	1 (3.33)	
Therapeutic failure	0	0	
Loss to follow-up	0	1 (3.33)	
Day 90			
Apparent cure	26 (86.67)	28 (93.33)	0.51^*a*^
Improvement	2 (6.67)	1 (3.33)	
No change	0	0	
Therapeutic failure	2 (6.67)	0	
Loss to follow-up	0	1 (3.33)	
Day 210			
Definite cure	24 (80.00)	28 (93.33)	0.21^*a*^
Therapeutic failure at day 90	2 (6.67)	0	
Therapeutic failure	3 (10.00)	0	
Loss to follow-up	1 (3.33)	2 (6.67)	

a Fisher exact test.

### Safety and adverse events.

At least one adverse event (AE) was observed in 56% of participants; no differences in the frequency of AEs were detected between study groups (*P* = 0.29). Eighty-nine percent of clinical adverse events were mild (grade 1); no reported events were classified as serious. In no case did intolerance require interruption of treatment. The most frequent AE were nausea, vomiting, abdominal pain, and mildly increased creatinine levels (Table 4). AEs were more frequently reported during weeks 1 and 2 of treatment (median, day 9; IQR, 1 to 19 days).

**TABLE 4:** Adverse events

Type of adverse event	No. (%)		*P* value
	Children (*n* = 30)	Adults (*n* = 30)	
Any^*a*^	15 (50)	19 (63)	0.29^*c*^
Vomiting	8 (26.7)	9 (30)	0.77^*c*^
Nausea	1 (3.3)	6 (20)	0.10^*b*^
Dizziness	2 (6.7)	4 (13.3)	0.67^*b*^
Abdominal pain	2 (6.7)	3 (10)	1.00^*b*^
Headache	1 (3.3)	3 (10)	0.61^*b*^
Fever	3 (10)	0	0.23^*b*^
Increased creatinine levels			
Grade 1 (>ULN^*d*^, ≤1.5 × ULN)	4 (13.3)	6 (20)	0.48^*c*^
Grade 2 or higher (>1.5 ULN)	0	0	
Other	7 (23.3)	10 (33.3)	0.56^*c*^

a Individuals presenting with at least one adverse event over the total of individuals in each study group.

b χ^2^ test.

c Fisher exact test.

d ULN, upper limit of normal.

## DISCUSSION

This pharmacokinetic study established that treatment of patients with CL using the current dosing regimen of 2.5 mg/kg/day results in lower systemic and intracellular exposure to miltefosine in children than in adults, consistent with results of PK modeling based on secondary data in children with VL (14, 15, 20, 21). We have recently shown noninferiority of miltefosine compared with meglumine antimoniate for treatment of cutaneous leishmaniasis in children 2 to 12 years of age (13), supporting its usefulness for treatment of the pediatric population. Considering its demonstrated efficacy, lower toxicity, and advantages of administration, miltefosine has been recommended for treatment of CL caused by *L*. (*V*.) panamensis and Leishmania (Viannia) guyanensis (22), which are prevalent in Central and South America and are frequently associated with transmission in the domestic setting where children are exposed (18).

Dose selection for miltefosine in the treatment of children has been based on efficacy studies conducted in adult populations and linear extrapolation of dose based on body weight. Descriptive PK studies have often concluded that linear dose scaling is not appropriate for children given their more rapid drug clearance (23, 24), as observed for antimony treatment of pediatric CL (3). Recent insights in PK suggest that linear dosing extrapolations result in lower systemic drug exposure in children due to the allometric principles underlying the relationship between metabolism and body size, as demonstrated for various other drugs (25).

Clinical responses provided evidence that lower exposure to miltefosine in children with CL is likely to affect therapeutic outcome, as evidenced by the occurrence of failures only in children ≤7 years of age (2, 4, 6, and 7 years). Future population-based compartmental modeling methods may allow more precise predictions of total exposure in plasma and peripheral blood mononuclear cells (PBMCs) in these patients and reveal an exposure-response relationship. We have previously proposed allometric dosing of miltefosine for VL patients, aiming to overcome differences in exposure observed by modeling of PK in children and adults (14). The lower exposure to miltefosine based on actual drug concentrations in these pediatric CL patients and their higher frequency of treatment failure compel evaluation of allometric dose scaling in the treatment of children with CL.

In addition to PK differences, other patient characteristics influence the outcome of treatment in CL patients (26–28). In our patient population, children presented a significantly shorter duration of disease at diagnosis than adults. Duration of disease of <1 month has been associated with increased risk of treatment failure with antimony (28–30) and may be an important covariate in the analysis and interpretation of therapeutic responsiveness. Exploratory analyses of patient and parasite characteristics identified younger age as a potential risk factor for therapeutic failure. Consistently, age <12 years was associated with relapse following miltefosine treatment of Nepalese children with VL (21), as was the case for 2- to 14-year-old children treated for VL in both India and Nepal (20). Similar findings have also emerged with meglumine antimoniate treatment, in which children <7 years of age presented a significantly lower response rate to this drug than to miltefosine (57.1% versus 89.9%) (13), supporting the participation of age-related factors in the therapeutic response of pediatric CL.

The *C*_max_ values of miltefosine in our study population, both children and adults, were similar to those reported in prior clinical studies from which PK were modeled (14, 31). However, additional PK parameters, such as AUC, *T*_max_, and *t*_1/2_, could not be compared, primarily because sampling schemes were not designed to generate concentration-time curves or these parameters were not reported.

This study provides the first intracellular PK data for miltefosine, which showed that intracellular drug concentrations tracked plasma concentrations, leading to similar proportional trends in intracellular and plasma PK parameters. However, a >2-fold higher concentration of miltefosine in the intracellular compartment indicated intracellular accumulation, which could be important to efficacy, since the observed concentrations exceeded the previously reported 50% effective concentration (EC_50_) for clinical strains of Leishmania (Viannia) (19).

Drug resistance was evidently not a defining factor in the outcome of treatment of these PK study participants. *In vitro* drug susceptibility did not differ among strains from patients who responded to or failed treatment. Similarly, miltefosine susceptibility did not differ for promastigotes of L. donovani strains from VL patients who failed or responded to treatment (21), underscoring the contribution of host factors in clinical outcome. Although parasite numbers declined, parasite burden was not indicative of clinical outcome, further substantiating that clinical resolution of CL is contingent not upon parasite elimination but rather on control of the infection, which has been shown to persist at least 90 days after completion of treatment in this study and indefinitely in previous studies (32). Perceived discrepancies between self-reported adherence and *in vivo* drug concentrations illustrate the challenge of assessing and achieving compliance for self-administered treatment (33). Patient-centered implementation strategies for miltefosine are necessary to optimize adherence and effectiveness of treatment.

Our PK data, and previous modeling studies of miltefosine in children with VL, consistently support the rationale for allometric dosing in children. Miltefosine has been suggested as first-line treatment for children with CL (13). Oral administration could allow home-based supervision of treatment, thereby facilitating access and adherence. However, dosing in children must be optimized to ensure adequate systemic exposure, effectiveness, and preservation of the useful life span of miltefosine.

## MATERIALS AND METHODS

### Ethics statement.

This study was approved and monitored by the institutional review board for ethical conduct of research involving human subjects of Centro Internacional de Entrenamiento e Investigaciones Médicas (CIDEIM) and the Colombian National Institute for Food and Drug Safety (INVIMA) and followed international guidelines. All individuals participated voluntarily, providing written informed consent. Legal guardians of pediatric patients provided written informed consent, and children aged ≥7 years provided written informed assent (ClinicalTrials.gov identifier NCT01462500).

### Study design.

An open-label trial was conducted to determine the pharmacokinetics of miltefosine in children and adults with CL. Sixty patients, 30 children and 30 adults, were enrolled from January 2012 through October 2013 at CIDEIM outpatient clinics in Cali and Tumaco, Colombia. Timing intervals of blood samples to determine the PK of miltefosine were based on published PK models generated with data from adult patients with Old World CL (34). Clinical and parasitological evaluations were undertaken to explore these variables in relation to PK parameters in children and adults.

Eligible participants were adults aged 18 to 60 years and children aged 2 to 12 years (weight, >10 kg) with parasitologically confirmed CL and availability for 6 months follow-up after treatment. Exclusion criteria were mucocutaneous disease, use of any antileishmanial drug during 6 months prior to diagnosis, medical history of cardiac, renal, or hepatic disease, menarche (females ≤12 years of age), pregnancy, and baseline values for hemoglobin, aspartate aminotransferase, alanine aminotransferase, creatinine, or serum urea nitrogen outside the normal range. In cases presenting borderline values, inclusion/exclusion was supported by clinical assessment. Contraception (Depo-Provera) was administered to women of reproductive age during treatment and throughout follow-up.

### Study interventions.

Patients received a nominal dose of 2.5 mg/kg/day miltefosine rounded to the nearest 10- or 50-mg capsule, with a maximum dose in adults of 150 mg/day, during 28 days. Doses received were registered in an individual patient diary, and the number of capsules and empty blisters were counted during follow-up visits to assess adherence. Peripheral blood samples (10 ml for adults and 3 ml for children) were collected for isolation of plasma and peripheral blood mononuclear cells (PBMCs) for quantification of miltefosine. Blood samples were collected at eight intervals over 7 months: pretreatment, after the first day of treatment (day 2), and days 15, 29, 60, 90, 120, and 210. A sampling window of ±7 days was accepted for all visits except pretreatment, day 2, and end of treatment (day 29) visits. Clinical laboratory tests performed at baseline were repeated at the end of treatment to monitor potential drug-related toxicity.

Adverse events (AEs), defined by clinical and laboratory criteria, were evaluated and recorded at every visit throughout treatment and follow-up. Severity of AEs was graded according to Common Toxicity Criteria of the National Cancer Institute, V.4 (35), and causality established using WHO-UMC criteria and the Naranjo algorithm (36). AEs were reported as possibly, probably, or definitely related to the study intervention.

Clinical response was evaluated at end of treatment and days 90 and 210. Cure was defined as complete reepithelialization and absence of inflammatory signs for all lesions. Definitive cure was established at day 210. Clinical failure was defined as incomplete reepithelialization and/or presence of induration, raised borders, or other evidence of inflammation of any lesion, reactivation of the original lesion(s), or appearance of new lesions during the follow-up period.

### PK analysis.

Miltefosine concentrations in plasma were determined by liquid chromatography-tandem mass spectrometry (LC-MS/MS) (37). Each analytical run included at least two calibration curves based on miltefosine standards injected at the beginning and end of the run. Quality control samples were included in two sets of low, medium, and high concentrations interspersed throughout the analytical run with study samples. Intracellular miltefosine was quantified from PBMCs as previously described (38). Total drug content was normalized to the number of nucleated cells per sample and concentrations estimated based on the average volume for a single PBMC (38). Noncompartmental PK analysis of concentration-time data was conducted using R (V.3.1.2) and ncappc (https://cran.r-project.org/web/packages/ncappc/index.html).

### Parasitologic assessments.

Lesion/scar aspirates and swab samples were obtained at diagnosis and days 29 and 90. Duplicate samples were obtained from the border of the most recent lesion for independent RNA and DNA extraction. DNA was extracted using the DNA blood and tissue kit (Qiagen), and RNA was detected using TRIzol followed by RNA cleanup with the RNeasy extraction kit (Qiagen). Purified RNA was treated with DNase I and eluted in a total volume of 35 μl. cDNA was synthesized from 10 μl total RNA using the high-capacity cDNA reverse transcription kit (Applied Biosystems). The quantity and quality of nucleic acids were evaluated using a NanoDrop 2000 spectrophotometer.

Leishmania kinetoplast DNA (kDNA) was amplified from samples by PCR using LV-B1 primers, followed by Southern blot hybridization (7). Amplification of the human GAPDH (glyceraldehyde-3-phosphate dehydrogenase) gene was employed to confirm sample quality (32). Live parasites and parasite burden were estimated by quantitative reverse transcription-PCR (qRT-PCR) of Leishmania 7SLRNA (5), calculated by absolute quantitation and normalized to the number of human cells in the sample determined by cyclophilin B expression. Real-time detection was achieved using SYBR green (Applied Biosystems) on a Bio-Rad CFX-96 platform. For kDNA-positive samples below the limit of detection by 7SLRNA qRT-PCR, a maximum-likelihood estimate of 0.0001 parasite per reaction was calculated (32). Qualitative assessment of parasite persistence at any given time point was based on detection of kDNA or 7SLRNA in at least one sample.

Leishmania was isolated by culture of tissue fluid obtained by needle aspiration of cutaneous lesions. Parasites were identified using subgenus- and species-discriminating monoclonal antibodies (39). Drug susceptibility of intracellular parasites was estimated after exposure to 16 μM miltefosine as described previously (19).

### Statistical analysis.

Data were verified by double entry prior to analysis. The Shapiro-Wilk test was used to assess the distribution of continuous data. Differences in variance of quantitative data were estimated using Mann-Whitney and *t* tests. Differences in proportions were determined using χ^2^ or Fisher exact tests. Relationships between therapeutic outcome and Leishmania species, parasite burden, drug susceptibility of isolated strains, *C*_max_, AUC_d0–29_ in plasma and PBMCs, patient age, and size and number of lesions were explored using logistic regression. Analyses were performed using Stata-14.

## Supplementary Material

Supplemental material
