# Supplementary material for: Pharmacokinetics of Miltefosine in Children and Adults with Cutaneous Leishmaniasis
Source: Antimicrob Agents Chemother. 2017 Feb 23;61(3):e02198-16. doi: 10.1128/AAC.02198-16 (PMC5328512; doi:10.1128/AAC.02198-16)
Supplement: Supplemental material [file supp_61_3_e02198-16__index.html]

Pharmacokinetics of Miltefosine in Children and Adults with Cutaneous Leishmaniasis — Supplemental material 

# Pharmacokinetics of Miltefosine in Children and Adults with Cutaneous Leishmaniasis

## Supplemental material

- Supplemental file 1 -

  Supplemental Table S1

  PDF, 142K
