# Supplementary material for: Pharmacokinetics of Miltefosine in Children and Adults with Cutaneous Leishmaniasis
Source: Antimicrob Agents Chemother. 2017 Feb 23;61(3):e02198-16. doi: 10.1128/AAC.02198-16 (PMC5328512; doi:10.1128/AAC.02198-16)
Supplement: Supplemental material [file AAC.02198-16_zac003175929s1.pdf]

Supplementary table 1. Compliance with protocol visits

| Day of visit | Planned time window | Visit attendance (n) | Observed time window median (IQR) | Comments |
|---|---|---|---|---|
| 0 | 0 | 60 | 0 (0 – 0) | |
| 1 | 0 | 60 | 1 (1 – 1) | |
| 15 | ± 7 | 59 | 15 (14 – 16) | One participant presented outside of sampling window (at day 25) |
| 29 | 0 | 59 | 29 (29 – 29) | Six patients from Tumaco presented outside the sampling window (at days 30-32). One patient presented at day 28. |
| 60 | ± 7 | 58 | 57 (56 – 58) | Two participants presented outside sampling window (at day 67) |
| 90 | ± 7 | 59 | 87 (86 – 89) | Three patients presented outside sampling window (at days 98, 103 and 106) |
| 120 | ± 7 | 53 | 117 (116 – 120) | One participant presented outside sampling window (at day 134) |
| 210 | ± 7 | 56 | 208 (206 – 210) | Seven participants presented outside sampling window (at days 222 – 243, one at day 287) |
